# Trends in hospitalization for pediatric hip arthroplasty: an epidemiological Nationwide study in Italy from 2001 to 2015

**DOI:** 10.1186/s12887-022-03302-5

**Published:** 2022-04-29

**Authors:** Umile Giuseppe Longo, Rocco Papalia, Sergio De Salvatore, Laura Ruzzini, Ilaria Piergentili, Giuseppe Salvatore, Vincenzo Candela, Vincenzo Denaro

**Affiliations:** 1 Research Unit of Orthopaedic and Trauma Surgery, Fondazione Policlinico Universitario Campus Bio-Medico, Via Alvaro del Portillo, Roma, 200 - 00128 Italy; 2 Research Unit of Orthopaedic and Trauma Surgery, Department of Medicine and Surgery, Università Campus Bio-Medico di Roma, Via Alvaro del Portillo, Roma, 21 - 00128 Italy

**Keywords:** Hip arthroplasty, Pediatric, Hip disorder, Epidemiology, Children, Hip replacement

## Abstract

**Background:**

The epidemiology of Pediatric Hip Arthroplasty (PHA) is unclear. Prevalence of PHA in Europe was reported in Scandinavian registries, but data on this procedure are not described in other countries. Therefore, it is challenging to redact a complete and valid epidemiological report on PHA in Europe. Nevertheless, national health statistics for PHA are helpful for an international audience, as different treatments are reported between countries. Moreover, sharing national statistics and correlating those to other countries’ protocols could be helpful to compare outcomes for different procedures internationally. The principal purpose is to evaluate the yearly hospital admission for PHA in Italy.

**Methods:**

Data of this study were collected from the National Hospital Discharge Reports (SDO) reported at the Italian Ministry of Health.

**Results:**

From 2001 to 2015, 770 PHA hospitalizations were performed in Italy, with an incidence of 0.5 procedures for every 100,000 pediatric Italian inhabitants. The average age of patients was 15.2 ± 4.6 years. The mean length of days of hospitalization was 10.9 ± 8.6 days. The majority of patients were male of 15–19 years old age group. A progressive decrease in days of hospitalizations was found during the years of the study.

**Conclusions:**

In Europe, the incidence of hospital admission for PHA is not fully described. There is a lack of consensus on the best type of surgery to perform on young patients. Epidemiological studies are helpful to understand the national variation of a specific surgical procedure and compare them with other countries.

## Background

Osteoarthrosis is the leading cause of 90% of primary hip arthroplasty in the adult population [1]. Slipped capital femoral epiphysis (SCFE), Perthes’ disease and Developmental Hip Dysplasia (DDH) constitute the most common causes of Pediatric Hip Arthroplasty (PHA) [2]. Pediatric hip disorders also include end-stage juvenile arthritis, osteonecrosis and tumours [3]. These conditions could lead to degenerative joint deformity and osteoarthrosis, requiring an early hip replacement [4]. The hip arthroplasty could be a standard hip arthroplasty or hip resurfacing, depending on the surgeon’s decision and the deformity [5]. The epidemiology of PHA is unclear, and few studies reported 2000 to 6000 procedures per year performed in the United States in patients under 25-years-old [1]. The prevalence of PHA in Europe is reported only in Scandinavian registries. The Norwegian arthroplasty registry reported data on 98% of all hip replacements in the last 30 years [6] among which 9% of arthroplasties were performed in the pediatric population [2, 7]. Due to the lack of data on hospitalization for PHA in Europe, it is difficult to estimate the real incidence of this procedure.

This study was conducted from 2001 to 2015, based on official data sources as hospitalization records. National health statistics for PHA are helpful for an international audience, as different protocols and treatments are reported between countries (type of surgery performed, mean age at the time of surgery, diagnosis and outcomes). Moreover, sharing national statistics and correlating those to other countries’ protocols, could be helpful to compare outcomes for different procedures internationally.

This paper aimed to evaluate the yearly number of hospital admission and characterize the patients who underwent PHA in Italy.

## Methods

Data of this study were collected from the National Hospital Discharge Reports (SDO) reported at the Italian Ministry of Health during the years of this paper (2001–2015). In Italy, the National Health Service (NHS) provides healthcare to all residents. The regional authorities are responsible for organizing and managing the healthcare services delivered through local structures (public and private accredited providers). Official data on all hospitalizations are collected by hospitals and local healthcare structures, entered into structured data files, and periodically sent to the Ministry of Health. Therefore, the ICD and “procedure codes” are reliable, and the National Hospital Discharge Reports are validated [8, 9]. These official data are anonymous and describe the patient’s age, sex, days of stay, diagnoses and procedures [10]. Population data were obtained from the National Institute for Statistics (ISTAT) for each year [9]. Hip arthroplasty was defined by the International Classification of Diseases, Ninth Revision, Clinical Modification (ICD-9-CM) 81.51 for PHA. The ICD-9-CM classification was used for both procedures and diagnoses. PHA included both standard total hip arthroplasties and hip resurfacing prostheses. ICD-9 did not allow to distinguish between the type of prosthesis. It was decided to analyze hip replacement cases only for pediatric patients (aged 0–19 years old).

### Statistics

Descriptive statistical analysis was used to estimate the yearly number of PHA, the percentage of males and females, the average age, days of hospitalization, primary diagnoses and primary procedures performed in Italy. Means, standard deviations, medians and interquartile ranges (IQR) for continuous variables and frequencies and percentages for categorical variables were computed. Incidence rates were calculated using the annual pediatric population size obtained from ISTAT. Poisson regression was used to evaluate if the PHA annual incidence increased or decreased with increasing or decreasing age or calendar year. The Statistical Package for Social Sciences (SPSS) version 26 (Armonk, NY: IBM Corp) was used for this data analysis. The graphs were performed using Excel Microsoft software (2019).

## Results

### Incidence of PHA hospitalization

**Fig. 1:**
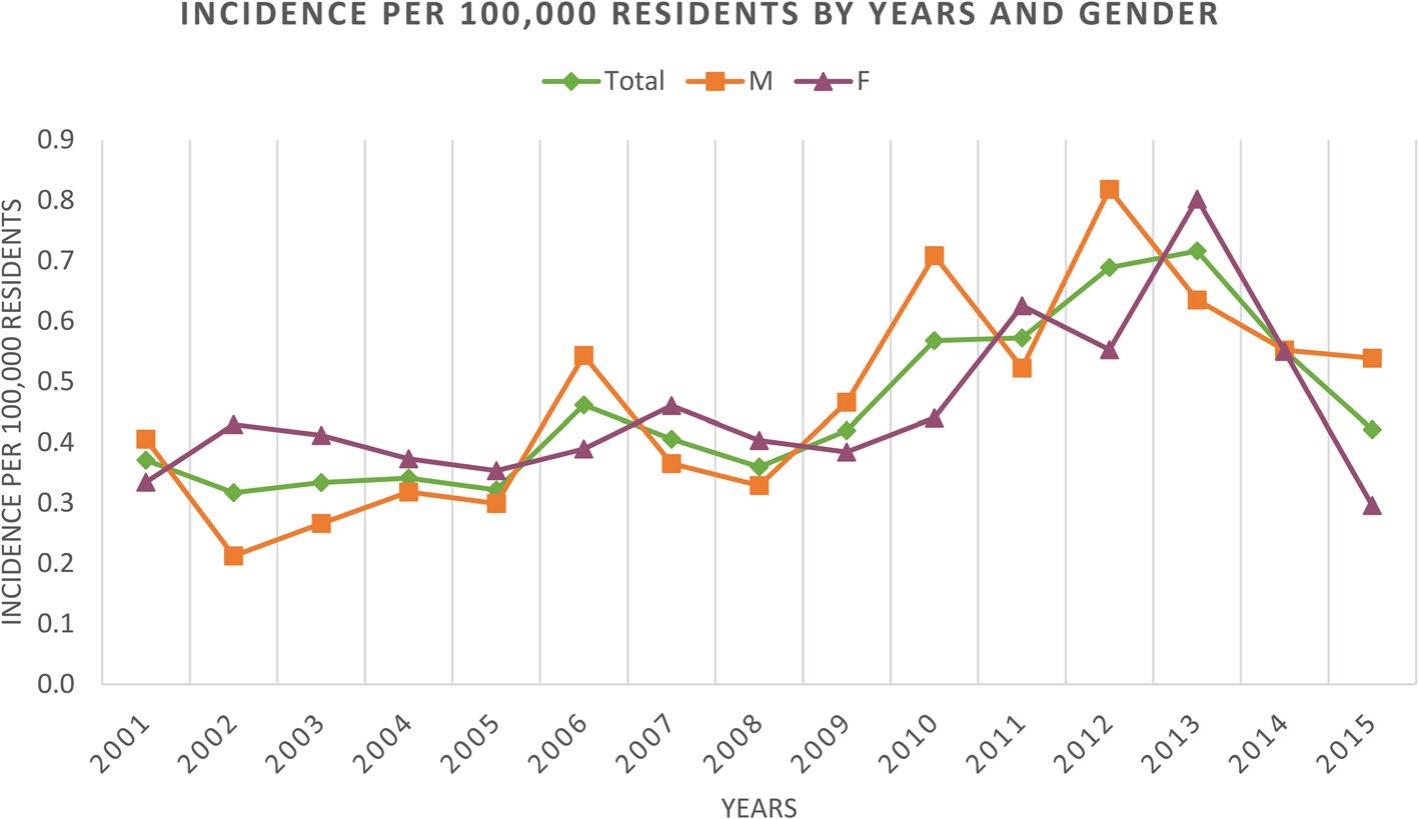
Incidence of PHAs per 100,000 residents by 2001 to 2015

During the 15-year study period, 770 hospitalizations for PHA were performed in Italy, with an incidence of 0.5 procedures for every 100,000 Italian inhabitants up to 19 years old, both for females and males. Overall and in female young patients, from 2001 to 2013, the incidence of PHA hospitalization increased from 0.4 to 0.7 procedures for every 100,000 young Italian residents, with a subsequent decrease until 2015 (*p* < 0.001) (Fig. 1). In male young patients, the incidence of PHA hospitalization increased from 2001 to 2006, decreased from 2006 to 2008, then increased from 2008 to 2012, with a subsequent decrease until 2015 (Fig. 1).

### Characteristics of the patients

**Fig. 2:**
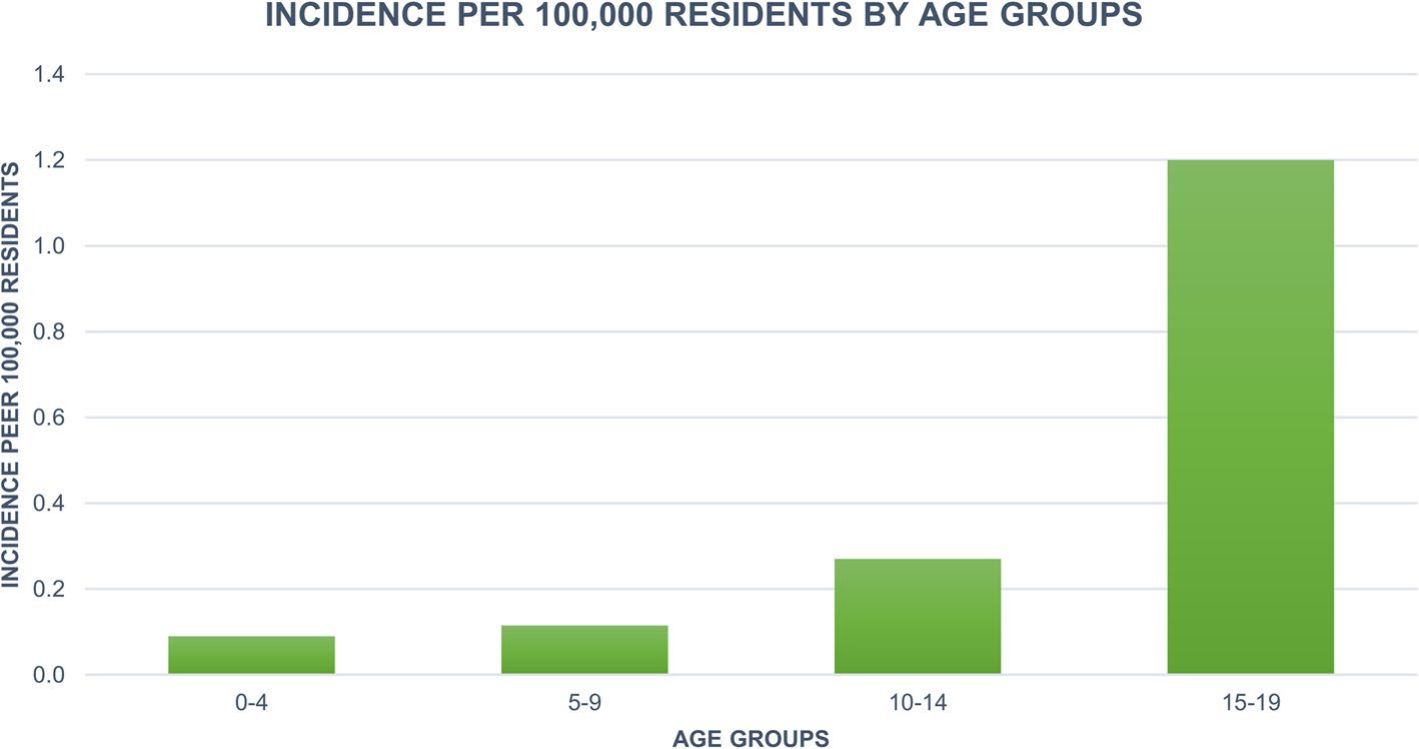
Incidence of PHAs by age groups

**Fig. 3:**
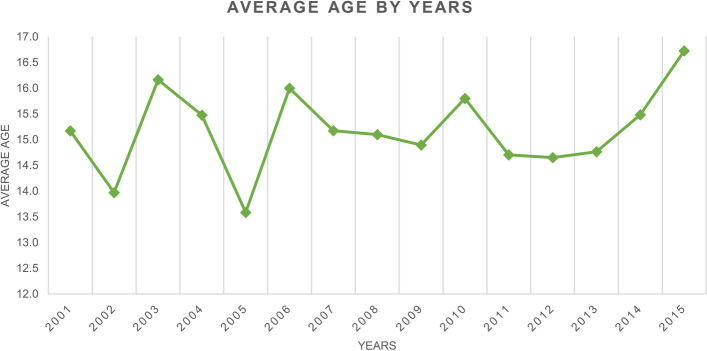
Average age over the years

Over the study period, the highest number of hospitalizations for PHA procedures (558 cases, with an incidence of 1.2 cases per 100,000 residents) was found in the 15- to 19-year age group (Fig. 2). While 122 hospitalizations were performed in patients aged between 10 and 14 years (incidence of 0.3 cases per 100,000 residents). 51 cases of PHA were reported in the 5–9 age group (with an incidence of 0.1 cases per 100,000 residents). 39 cases of PHA were recorded in the 0–4 age group (with an incidence of 0.1 cases per 100,000 residents). The average age of patients who underwent PHA was 15.2 ± 4.6 years, with an increasing trend in the last study years (Fig. 3). There is no statistically significant correlation between the trend of PHA annual incidence and the increasing age of patients (*p* = 0.988). Males represented the majority of patients undergoing PHA (males 52.1% and females 47.9%).

### Length of hospital stay

**Fig. 4:**
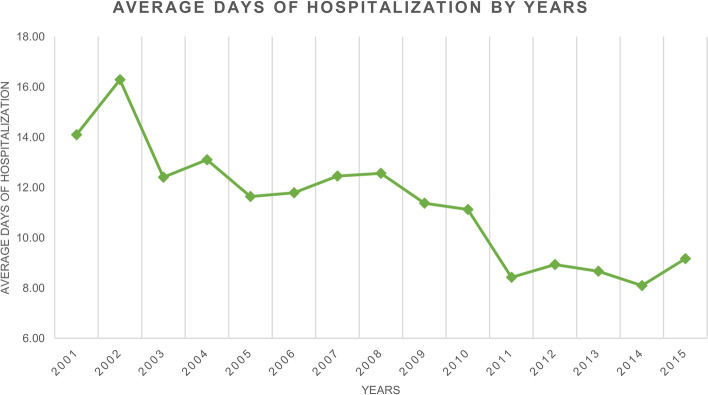
Average days of hospitalization over the years

**Fig. 5:**
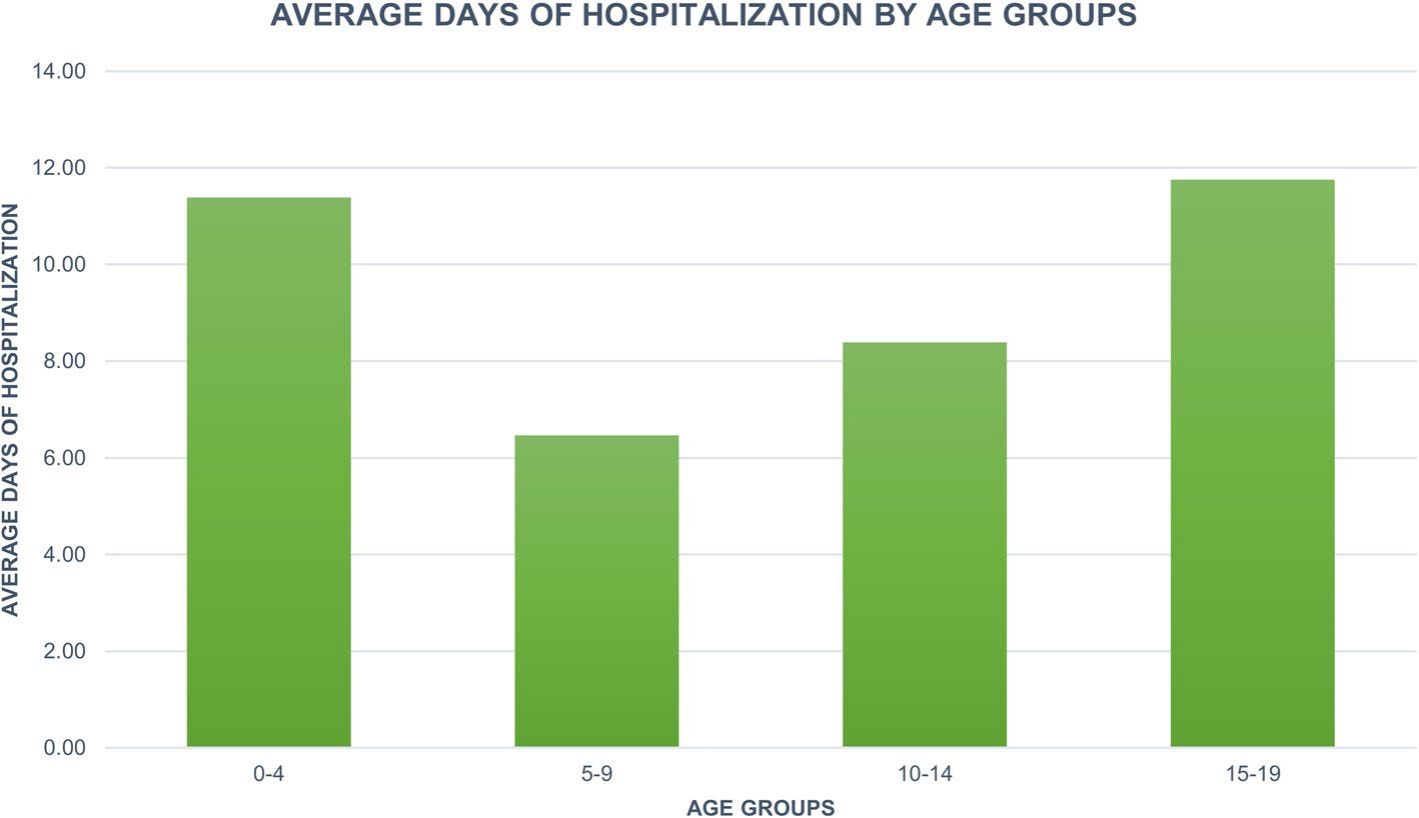
Average days of hospitalization by age groups

The mean length of days of hospitalization was 10.9 ± 8.6 days for PHA. Females and males had similar lengths of stay, the average was 10.8 ± 6.8 days and 10.9 ± 10 days; respectively. The median days of hospitalization were 9 (IQR = 8) in females and 9 (IQR = 7) in males. A general trend of decrease in days of hospitalization in both groups was reported (Fig. 4). Patients aged 15 to 19 had more days of hospitalization on average after PHA (11.8 ± 9.2 days) (Fig. 5).

**Fig. 6:**
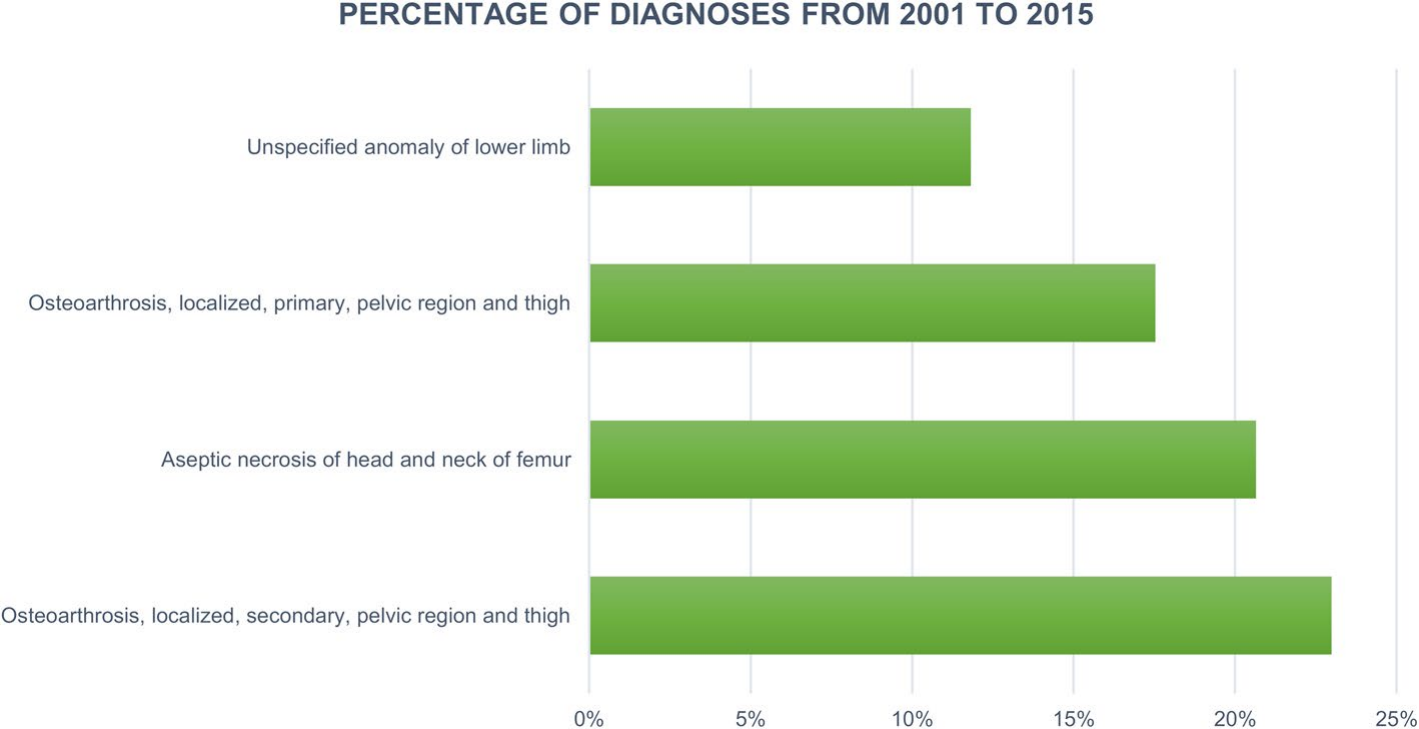
Percentage of diagnoses for PHAs hospitalizations from 2001 to 2015

During the 15-year study period, the main primary diagnoses in patients who underwent PHA were “Osteoarthrosis, localized, secondary, pelvic region and thigh” (23%, ICD-9-CM code: 715.25), “Aseptic necrosis of head and neck of femur” (20.6%, ICD-9-CM code: 733.42), “Osteoarthrosis, localized, primary, pelvic region and thigh” (17.5%, ICD-9-CM code: 715.15) and “Unspecified anomaly of lower limb” (11.8%, ICD-9-CM code: 755.60) (Fig. 6).

## Discussion

The aim of this study was to estimate the prevalence of PHA procedures in the Italian population under 19 years old. From 2001 to 2015, the mean incidence of PHA was 0.5 for every 100,000 inhabitants under 19 years old. In the last 2 years of the study (2014–2015) a mild decrease in incidence was reported, with a lower value in 2015. The majority of patients were male in the 15–19 years old age group.

Pediatric hip disorders include a broad spectrum of diseases that affect children and lead to joint deformity and osteoarthrosis [11]. In late diagnosis and treatment, these conditions lead to severe deformities that require a hip replacement. The incidence of PHA is not fully understood, especially in Europe, reaching 9% of hip arthroplasties in Norwegian registries. In the study of Halvorsen et al. [7] were reported 881 PHAs between 1995 and 2016 in Denmark (253 PHAs), Finland (171 PHAs), Norway (207 PHAs) and Sweden (250 PHAs). The male-female ratio was close to 1:1, except in Sweden, where the ratio was almost 1:2 [7].

The most common condition that leads to PHA is Perthes’ disease, followed by SCFE [12–14]. Both diseases, especially in case of late diagnosis and treatment, generate a collapse of the femoral head with consequent hip osteoarthritis and loss of function. The most common symptoms are decreasing in Range of Motion (ROM), shortening the affected limb, pain and limping localized to the hip, groin, thigh or knee. Pediatric hip disorders also include end-stage juvenile arthritis, osteonecrosis and tumours, but they usually require hip replacement in rare cases during pediatric age [11].

A precise and rapid diagnosis is challenging due to the differential causes of hip pain in young patients [15]: apophyseal avulsion fracture of the anterosuperior and anteroinferior iliac spine; apophysitis of the anterosuperior and anteroinferior iliac spine; transient synovitis; fracture; septic arthritis; adductor muscle strain [16, 17]. The most frequent problems in PHA are prosthetic loosening, osteolysis and implant life span [18]. Despite the indication for hip replacement in pediatric patients is the same as in adults (persistent pain, loss of function and decreased ROM) [5, 19], considering the higher risk in young patients, caution needs to be placed before surgery.

The indication for standard total hip arthroplasty or hip resurfacing arthroplasty is similar: patients unresponsive to conservative treatment with end-stage osteoarthritis [20]. Hip resurfacing arthroplasty is mostly indicated in the post-traumatic arthritis of pediatric male patients [21]. Otherwise, although the conservative approach of hip resurfacing should be considered in young patients, in many cases of pediatric hip disorders, the altered anatomy of the joint should lead to deciding for a total hip replacement [5]. In case of severe DDH, the acetabular bone stock is insufficient, making the resurfacing more difficult without the possibility to use screw fixation. Although the cups of resurfacing prostheses use peripheral screw fixation to improve the press-fit of the monoblock cup, the efficacy of these elements is not thoroughly investigated [5, 22]. Moreover, young patients with high lower limb discrepancy could take more benefits with a total hip replacement, rather than a resurfacing prosthesis. In patients with severe Perthes’ disease or SCFE, hip resurfacing arthroplasty is contraindicated due to the altered anatomy and the insufficient bone stock of the femoral head [23]. Another critical limit of hip resurfacing arthroplasty is the metal ion absorption, but recent progress in metallurgy has drastically reduced the risk [24].

To date, no international consensus on the proper surgical treatment (standard PHA versus hip resurfacing) was found in the literature [5, 25–27]. Therefore, further high-quality clinical trials are required to obtain specific results. International epidemiological studies could provide data on incidence, the procedure performed and outcomes among different surgical procedures.

A progressive decrease in days of hospitalizations was found. Moreover, patients aged between 15 and 19 years old reported higher hospitalisation days after PHA than the others.

### Limitations

This study has some limits. It was based on administrative data from different hospitals and macro-regions. The International Classification of Diseases 9 (ICD-9) was used for all the procedures reported. Moreover, it was impossible to distinguish unilateral vs bilateral PHA because ICD-9 did not fully code it. Furthermore, ICD-9 did not differentiate hip resurfacing from standard total hip arthroplasty. Moreover, it was not possible to compare hip arthroplasty to other key health conditions. Unfortunately, there is no information about other health conditions in the database provided by the Italian Ministry of Health. The information in the database were age, sex, days of hospitalization, ICD-9-CM code of the diagnosis and ICD-9-CM code of the procedure. Patients’ names had been replaced with codes to maintain privacy. Lastly, ICD-9 did not provide information on the pre-operatory symptoms and the clinical status of the patients; therefore, it would not be possible to provide this data and compare clinical outcomes.

## Conclusions

In Europe, the incidence of hospital admission for PHA is not fully described. The results of this study showed an incidence of PHA in the Italian pediatric population of 0.5 cases/100,000 inhabitants. The majority of patients were male in the 15–19 years old age group. A progressive decrease in days of hospitalizations was found during the years of the study. There is a lack of consensus on the best type of surgery to perform in young patients. Epidemiological studies are helpful to understand the national variation of a specific surgical procedure and compare them with other countries.

## Data Availability

The datasets used and/or analyzed during the current study are not publicly available due on our policy statement of sharing clinical data only on request but are available from the corresponding author on reasonable request. The access to the database is on request. All data were obtained by the Direzione Generale della Programmazione Sanitaria—Banca Dati SDO of the Italian Ministry of Health.

## Abbreviations

SCFE: Slipped capital femoral epiphysis
DDH: Developmental Hip Dysplasia
PHA: Pediatric Hip Arthroplasty
SDO: National Hospital Discharge Reports
NHS: National Health Service
ICD-9-CM: International Classification of Diseases, Ninth Revision, Clinical Modification
SPSS: Statistical Package for Social Sciences
